# Effect of EMG-triggered neuromuscular electrical stimulation with bilateral arm training on hemiplegic shoulder pain and arm function after stroke: a randomized controlled trial

**DOI:** 10.1186/s12984-017-0332-0

**Published:** 2017-11-28

**Authors:** Li-Ling Chuang, You-Lin Chen, Chih-Chung Chen, Yen-Chen Li, Alice May-Kuen Wong, An-Lun Hsu, Ya-Ju Chang

**Affiliations:** 1 Department of Physical Therapy and Graduate Institute of Rehabilitation Science, College of Medicine, Chang Gung University, Taoyuan, Taiwan; 2 Healthy Aging Research Center, Chang Gung University, Taoyuan, Taiwan; 3 Department of Physical Medicine and Rehabilitation, Chang Gung Memorial Hospital, Linkou Medical Center, Taoyuan, Taiwan; 4 Physical therapy, Department of Physical Medicine and Rehabilitation, Chang Gung Memorial Hospital, Taoyuan, Taiwan; 5 Department of Physical Therapy, Mackay Memorial Hospital, Taipei, Taiwan; 6 Neuroscience Research Center, Chang Gung Memorial Hospital, Linkou Medical Center, Taoyuan, Taiwan; 7 No.259, Wenhua 1st Rd., Guishan Dist., Taoyuan, 33302 Taiwan

**Keywords:** Shoulder pain, Stroke rehabilitation, Electric stimulation therapy

## Abstract

**Background:**

Hemiplegic shoulder pain is a frequent complication after stroke, leading to limited use of the affected arm. Neuromuscular electrical stimulation (NMES) and transcutaneous electrical nerve stimulation (TENS) are two widely used interventions to reduce pain, but the comparative efficacy of these two modalities remains uncertain. The purpose of this research was to compare the immediate and retained effects of EMG-triggered NMES and TENS, both in combination with bilateral arm training, on hemiplegic shoulder pain and arm function of stroke patients.

**Methods:**

A single-blind, randomized controlled trial was conducted at two medical centers. Thirty-eight patients (25 males and 13 females, 60.75 ± 10.84 years old, post stroke duration 32.68 ± 53.07 months) who had experienced a stroke more than 3 months ago at the time of recruitment and hemiplegic shoulder pain were randomized to EMG-triggered NMES or TENS. Both groups received electrical stimulation followed by bilateral arm training 3 times a week for 4 weeks. The primary outcome measures included a vertical Numerical Rating Scale supplemented with a Faces Rating Scale, and the short form of the Brief Pain Inventory. The secondary outcome measures were the upper-limb subscale of the Fugl-Meyer Assessment, and pain-free passive shoulder range of motion. All outcomes were measured pretreatment, post-treatment, and at 1-month after post-treatment. Two-way mixed repeated measures ANOVAs were used to examine treatment effects.

**Results:**

Compared to TENS with bilateral arm training, the EMG-triggered NMES with bilateral arm training was associated with lower pain intensity during active and passive shoulder movement (*P* =0.007, *P* =0.008), lower worst pain intensity (*P =* 0.003), and greater pain-free passive shoulder abduction (*P* =0.001) and internal rotation (*P* =0.004) at follow-up. Both groups improved in pain at rest (*P* =0.02), pain interference with daily activities, the Fugl-Meyer Assessment, and pain-free passive shoulder flexion and external rotation post-treatment (*P* < 0.001) and maintained the improvement at follow-up (*P* < 0.001), except for resting pain (*P* =0.08).

**Conclusions:**

EMG-triggered NMES with bilateral arm training exhibited greater immediate and retained effects than TENS with bilateral arm training with respect to pain and shoulder impairment for chronic and subacute stroke patients with hemiplegic shoulder pain.

**Trial registration:**

NCT01913509.

## Background

### Introduction

Hemiplegic shoulder pain is a common complication following stroke that restricts shoulder mobility and may interfere with rehabilitation [1]. Initial post-stroke weakness and spasticity lead to shoulder instability and immobility, which can cause pain directly or place the capsule at risk for trauma, subsequently leading to pain [2]. The etiology of hemiplegic shoulder pain is multifactorial, including shoulder subluxation, spasticity in the pectoralis major and subscapularis, adhesive capsulitis, bursitis, tendonitis, and shoulder-hand syndrome [3]. Accordingly, a wide variety of current treatment regimens have been used, such as shoulder positioning, slings and support aids, strapping and taping, surgical interventions, triamcinolone acetonide injections, electrical stimulation, and so on [3, 4].

The most promising interventions for hemiplegic shoulder pain are surface or percutaneous neuromuscular electrical stimulation (NMES) and intraarticular corticosteroid injections [3, 4]. Although corticosteroid injections may give satisfactory results, potential side effects include post-injection flare and tendon rupture [5]. Percutaneous NMES requires invasive procedures to implant electrodes and poses the risk of electrode-related infections, which makes clinical implementation difficult [6, 7]. Given the potential adverse effects of percutaneous NMES, it is more conservative to use surface NMES for hemiplegic shoulder pain.

NMES and transcutaneous electrical nerve stimulation (TENS) are two widely used interventions to reduce pain in clinical practice [8]. The distinction between these two interventions is that NMES is used to produce muscle tetany to improve pain, whereas TENS is specifically used for pain relief at the sensory level, with no muscle contraction [7, 9]. Theoretically, NMES helps contract and strengthen muscles to prevent disuse atrophy, relax muscle spasm, increase blood circulation and nutrition of the muscles, and reeducate muscles [2, 9, 10].

Eight controlled trials of surface NMES for hemiplegic shoulder pain have been reported [11–18]. The stimulation was applied over periods ranging from 4 weeks to 3 months. Some of these studies demonstrated that NMES not only reduced shoulder pain and subluxation [11, 12, 16, 18], but also improved shoulder abduction and arm function in patients with acute stroke [12, 18]. In contrast, Church et al. found no difference in upper-limb pain and arm function between NMES and placebo groups after acute stroke [13]. The authors proposed that surface NMES may interfere with upper-limb motor recovery by producing artificial proximal stimulation [13]. Wang et al. found that a NMES program significantly improved motor function for patients with acute stroke, but had no effect on pain-free passive external shoulder rotation [17]. However, significant differences in the severity of baseline disability between groups made their interpretations uncertain. The disparity in these studies could be due to many factors, among which are the heterogeneity of study populations, interventions, and outcome measures used; inconsistent definitions of hemiplegic shoulder pain [19, 20]; inclusion of subjects without shoulder pain [15, 21]; and lack of an appropriate guide for treatment modality selection [3]. Moreover, the existence of different treatment doses may have generated bias in some studies, in which the experimental group received NMES therapy in addition to conventional rehabilitation and had more attention to their shoulder than the control group [12, 14, 15, 18]. Finally, most of the studies did not examine the effects of NMES beyond the termination of treatment.

Recently, surface NMES has been applied in combination with other approaches to create a task-specific training paradigm [10]. Chan et al. found that surface NMES with bilateral arm training produced significant improvements in arm function and active wrist extension when compared with placebo stimulation with bilateral arm training [22]. Cauraugh and Kim combined electromyography (EMG)-triggered NMES with bilateral arm training and showed improved motor control in a bimanual task [23, 24]. De Kroon et al. found EMG-triggered NMES may be more effective than non-triggered stimulation in producing improvements in motor control of the hemiparetic arm [6]. EMG-triggered NMES is unique in that it requires subjects’ active participation in the training via cognitive intent to trigger electrical stimulation and activate the corresponding NMES-induced muscle contraction [6, 10]. Accordingly, it would be interesting to investigate the effects of EMG-triggered NMES with bilateral arm training on hemiplegic shoulder pain. However, there is a lack of research on the combined effects of EMG-triggered NMES and functional training to identify the most effective strategy for reducing hemiplegic shoulder pain and improving arm function. Therefore, the primary aim of this study was to investigate the effects of EMG-triggered NMES combined with bilateral arm training on hemiplegic shoulder pain and arm function, as compared with TENS combined with bilateral arm training, in subacute and chronic stroke patients. The secondary aim was to evaluate the retention of the treatment effect at 1 month after the intervention. The primary hypothesis was that NMES combined with bilateral arm training would improve pain and arm function more than TENS combined with bilateral arm training, and the secondary hypothesis was that the therapeutic effects of NMES combined with bilateral arm training would be retained more than those of TENS combined with bilateral arm training.

## Methods

### Participants

Subjects were recruited from two medical centers (Mackay Memorial Hospital and Chang Gung Memorial Hospital). The recruitment criteria were as follows: (1) first-ever stroke with onset >3 months prior at time of recruitment; (2) at least mild intensity of hemiplegic shoulder pain with activity in the past 7 days (Numerical Rating Scale score ≥ 1); (3) no other neurological disorders, such as Parkinson’s disease, epilepsy, multiple sclerosis, etc.; (4) adequate cognitive ability (Mini-Mental State Examination score ≥ 24). The exclusion criteria were (1) contraindications for electrical stimulation (e.g., metal implants, cardiac pacemaker); (2) pre-existing pathology of the shoulder, such as rotator cuff injury or tendonitis, frozen shoulder, etc.; (3) participation in any experimental rehabilitation or drug studies during the study period; (4) change of pain medication during the study period; (5) treatment of upper limb spasticity, including botulinum toxin injection or neurolytic or surgical procedures; (6) aphasia; and (7) severe cognitive deficits.

The study was approved by the Institutional Review Boards of the participating sites. All participants provided written informed consent and were informed of the study’s purpose, the process, and their right to withdraw from the study at any time.

### Study design and randomization

**Fig. 1:**
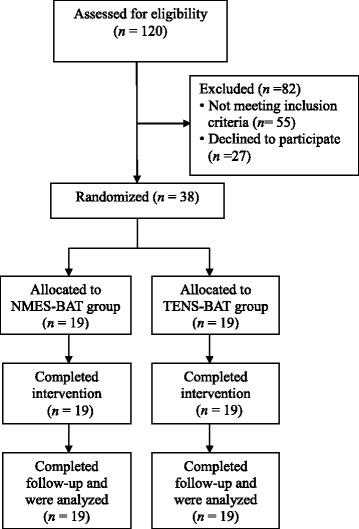
Flow chart of participants who enrolled in and completed the study. Abbreviations: NMES, neuromuscular electrical stimulation; TENS, transcutaneous electrical nerve stimulation; BAT, bilateral arm training

This study was a single-blind, randomized controlled trial. The ClinicalTrials.gov identifier number is NCT01913509. The Consolidated Standards of Reporting Trials (CONSORT) flow chart is presented in Fig. 1. After obtaining written informed consent, the eligible participants were randomly assigned to one of two training groups according to a computer-generated list, with stratification by side of brain lesion. The stratification was performed since recovery profile is influenced by the initial stage, such as side of lesion and motor severity. The two training groups included: (1) EMG-triggered NMES with bilateral arm training; (2) TENS with bilateral arm training. The allocation sequence was carried out by a research assistant and concealed in opaque, sealed envelopes. Participants were blinded to the type of treatment.

### Interventions

The participants received the interventions for 12 sessions (3 days/week for 4 weeks). Participants in the experimental group received EMG-triggered NMES and those in the control group received TENS for 20 min. After the EMG-triggered NMES or TENS, all participants received 20 min of bilateral arm training, including bilateral arm raises, bilateral arm reaching forward, bilateral shoulder abduction, and bilateral shoulder horizontal abduction at pain-free range. The number of repetitions of the bilateral arm training exercises was based on each individual’s capability and was gradually increased throughout the treatment sessions.

A portable, two-channel neuromuscular stimulator (PAS System™ GD601: OG GIKEN Company, Okayama, Japan) with trigger mode was used to deliver EMG-triggered NMES for the experimental group. Trigger mode was used to start the low frequency output when EMG feedback was detected. The system uses a three-electrode format and EMG feedback detection to allow EMG-triggered electrical stimulation of the target muscles (i.e., supraspinatus and posterior deltoid). The gain dial was used to adjust the sensitivity of EMG feedback detection and the EMG monitor lit up when EMG feedback was detected. When EMG feedback over the level set with the gain dial was detected, the output voltage was gradually increased. After a certain duration, the output voltage was gradually decreased to allow the muscle to return to a resting state. After the rest time passed, the next EMG feedback detection was enabled. The supraspinatus and posterior deltoid muscles were selected as the targets for treatment, as they are key muscles in maintaining correct shoulder alignment and providing stabilization of the shoulder joint [10, 25, 26]. Therapeutic electrical stimulation to the supraspinatus and posterior deltoid muscles has been shown to effectively reduce shoulder subluxation and pain, increase muscle force, and facilitate shoulder stability [11, 12, 14–18]. Electrode placement for the supraspinatus and posterior deltoid muscles was assisted by palpation, visual inspection of the muscles, and skin markings for the spine of the scapula and the acromion process. Two active electrodes were placed over the palpated muscle belly along the length of the muscle. Electrode placement for the supraspinatus was 1.5 cm superior to the midpoint of the spine of the scapula. Electrode placement for the muscle belly of the posterior deltoid was two fingerwidths inferior to the posterior margin of the acromion process [27]. Participants were instructed to initiate a voluntary isotonic contraction of the shoulder abductors and horizontal abductors with effort, respectively. Surface electrodes detected the EMG feedback signal at the target muscle and then the target muscle was electrically stimulated. A stimulation frequency of 30 Hz was used to generate a tetanized contraction, and the intensity was individually adjusted to produce significant muscle contraction within the maximum tolerance level. The range of intensities used to stimulate the muscles was 3–5 out of 10. The contraction-relaxation ratio of EMG-triggered NMES was adjusted progressively from 10/10 s to 30/10 s [15]. There were no adverse events, such as burns or skin allergic responses, during the study period.

The control group received TENS on the supraspinous fossa and posterior deltoid muscles of the painful shoulder, which was performed by a portable stimulator unit (SW320, Shining World Health Care Co., LTD., Taiwan) at a frequency of 30 Hz. TENS was applied using a similar treatment protocol, electrode placement, and stimulation frequency as the experimental group. According to the manufacturer’s instructions, the level of intensity was set from 1 through 5 at the highest comfortable setting but below the motor threshold, as the intensity setting varies individually. To find the electrode placement for the target muscles, the participants in the TENS group initiated a voluntary movement and received higher level of stimulation intensity at the beginning. Then the intensity of stimulation was adjusted gradually to lower level to the maximum tolerable sensory level without muscle contraction.

### Outcome measures

All outcomes were measured pretreatment, post-treatment, and 1 month after posttreatment. Hemiplegic shoulder pain can interfere with activities of daily living and subsequently lead to disability and poor functional recovery of the affected arm. To determine pain intensity and pain interference with daily activities, two pain measures (a vertical Numerical Rating Scale supplemented with a Faces Rating Scale [NRS-FRS] and the short form of the Brief Pain Inventory [BPI-SF]) were chosen as the primary outcome measures. Regarding the extent of upper-limb impairment and dysfunction, two measures (the upper-limb subscale of the Fugl-Meyer Assessment [FMA-UL] and pain-free passive shoulder range of motion) were chosen as the secondary outcome measures.

Pain intensity was measured on a 10-point vertical NRS with word anchors supplemented with the six facial expressions of the FRS, facilitating the scoring of pain. Participants were asked to rate the intensity of their hemiplegic shoulder pain at rest, and during active and passive range of motion of the affected shoulder. Shoulder pain was significantly more frequent in subjects with limitation of flexion, abduction, and external rotation of shoulder [28, 29]. For the assessment of shoulder pain during movement, immediately following the performance of active and passive shoulder range of motion, subjects were asked to mark the vertical NRS-FRS at the point that corresponded to the level of pain they experienced during shoulder flexion, abduction, and external rotation, respectively. The vertical NRS-FRS is a reliable measure of pain after stroke, with good test-retest reliability [30].

The BPI-SF is a 9 item questionnaire for the assessment of worst, least, average, and current pain intensity and the degree that pain interferences with daily activities on a 10 point scale [31]. The statement of question 3 of the BPI-SF was as follows: “Please rate your pain by marking the number that best describes your pain at its worst in the last 24 hours.” Question 3 of the BPI-SF was used to ask participants to rate the worst shoulder pain intensity in the past 24 h, with 0 being “no pain” and 10 being “pain as bad as you can imagine.” The statement of question 9 of the BPI-SF was as follows: “Mark the number that describes how, during the past 24 hours, pain has interfered with your general activity, mood, walking ability, normal work, relations with other people, sleep, and enjoyment of life, respectively.” Question 9 of the BPI-SF was used to estimate the degree to which shoulder pain interfered with daily life in the past week, with 0 being “no interference” and 10 being “interferes completely.” The BPI-SF has demonstrated good reliability and validity for clinical pain assessment across cultures and languages. A Chinese version of the BPI-SF was developed and proven to be a reliable and valid measure of both the severity and impact of pain among Taiwanese cancer patients [32, 33].

The FMA-UL was used to measure motor impairment [34]. The FMA-UL includes 33 items (total score: 0–66 points) and is scored on a 3-point ordinal scale (0 =cannot perform, 1 =performs partially, 2 =performs completely), with good reliability and validity [35]. The FMA-UL can be divided into 21 proximal (0–42 points) and 12 distal (0–24 points) items. A higher score on the FMA-UL indicates better motor function. Limited range of passive shoulder external rotation and abduction was correlated highly with hemiplegic shoulder pain [14, 16–18]. Motor impairment and pain were also assessed by measuring the pain-free passive range of motion of the hemiplegic shoulder using a clinical goniometer, where a loss of range indicated an increase in pain [36].

### Sample size calculation

The sample size calculation was performed with G*Power 3 (a statistical power analysis program) [37]. The effect size computation was based on Cohen’s d, with an expected effect of 0.2 to 0.5. Two-way mixed repeated measures ANOVA indicated that a total sample size of 28 was needed to reach 80% power to detect an interaction effect size of 0.25 at the 0.05 level of significance. The sample size requirement for each group in a 2-group study design was 14 subjects. With a potential 20% attrition rate, a total of 34 subjects were targeted for this study.

### Statistical analysis

The independent t-test and χ^2^ test were used to compare the baseline characteristics between groups. Descriptive statistics were presented using the mean (standard deviation) or median (range) for continuous variables, or numbers for categorical variables. The effect of the interventions on the primary and secondary outcomes was analyzed using 2 × 3 mixed repeated measures ANOVAs with group (i.e. NMES combined with bilateral arm training, TENS combined with bilateral arm training) as the between-subject factor and time (i.e. pretreatment, post-treatment, 1-month follow-up) as the within-subject factor. Effect sizes were calculated as partial eta squared (η^2^) for ANOVA results. Post hoc planned pairwise comparisons through t-tests, with Bonferroni correction for multiple comparisons, were performed when significant interactions or main effects for factors were observed. In addition, subgroup analysis was used to compare the changes in outcomes over time from pretreatment to post-treatment and follow-up in subjects with or without subluxation (i.e., NMES combined with bilateral arm training group without subluxation, NMES combined with bilateral arm training group with subluxation, TENS combined with bilateral arm training group without subluxation, TENS combined with bilateral arm training group with subluxation) by using ANOVAs. The significance level was set at 0.05. Statistical analyses were performed with SPSS 20.

## Results

This study was conducted from December 2013 to June 2017. Thirty-eight participants were randomized, 19 in NMES combined with bilateral arm training and 19 in TENS combined with bilateral arm training. No participant was lost to follow-up, and none were excluded after randomization, such that all 19 participants in each group were included in the final analysis according to their originally assigned groups. The baseline characteristics of the 38 participants are summarized in Table 1.

**Table 1:** Baseline characteristics of the participants

	NMES-BAT (*n* = 19)	TENS-BAT (*n* = 19)	*P* values
Age (mean years ± SD)	58.89 ± 11.93	62.61 ± 9.59	0.30
Gender (male: female)	13: 6	12: 7	0.73
Type of stroke (ischemic: hemorrhagic)	9: 10	9: 10	1.00
Side of hemiplegia (right: left)	10: 9	7: 12	0.52
Dominant hand (right: left)	18: 1	18: 1	1.00
Time since stroke (mean months ± SD)	31.89 ± 55.59	33.47 ± 51.94	0.93
Brunnstrom stage-Upper limb, median			
-Proximal part (range)	5 (3–5)	5 (4–5)	0.29
-Distal part (range)	5 (1–5)	5 (1–5)	0.70
Mini Mental State Examination	28.63 ± 1.61	28.74 ± 1.70	0.85
Shoulder subluxation^a^, n (%)	9 (47%)	8 (42%)	0.74

Abbreviations: *SD* indicates standard deviation; n, subgroup number, *NMES* neuromuscular electrical stimulation, *TENS* transcutaneous electrical nerve stimulation, *BAT* bilateral arm training

^a^Shoulder subluxation was defined as incorrect alignment between the scapula and the humerus, as compared with the unaffected shoulder

Nine subjects (47%) in the NMES combined with bilateral arm training group and 8 subjects (42%) in TENS combined with bilateral arm training group had shoulder subluxation, which was measured by palpation of the space between the acromion and the humeral head. The size of the space was quantified by how many fingers could be placed between the acromion and the humerus [38]. There was no statistically significant difference between the 2 groups at pretreatment. No harms or unintended effects were reported in either group.

### Effect of interventions on primary outcomes

**Fig. 2:**
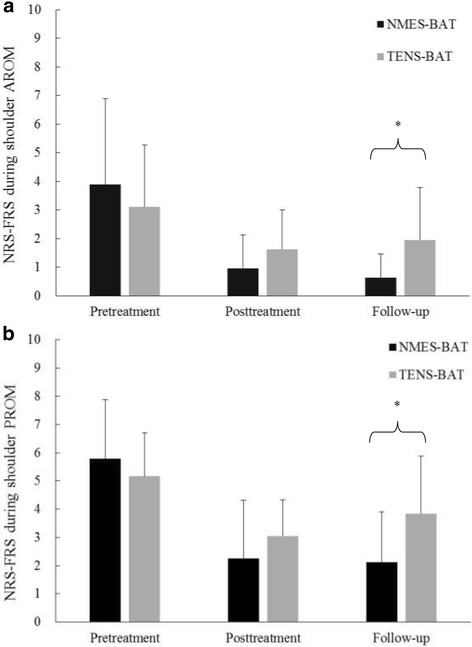
NRS-FRS scores during (**a**) shoulder active range of motion and (**b**) shoulder passive range of motion. * *P* < 0.05 between groups as indicated

Two-way mixed ANOVA revealed a significant time effect (F_1.17, 42.04_ =4.65, *P* =0.03, partial η^2^ =0.11, observed power =0.60), and no significant interaction and group effects (*P* > 0.05) on the vertical NRS-FRS for assessing pain at rest (Table 2). Both groups had significantly decreased shoulder pain at rest after the interventions (*P* =0.02). There was a significant interaction of time and group on the vertical NRS-FRS for assessing pain during active shoulder range of motion (F_1.45, 52.22_ =5.84, *P* =0.01, partial η^2^ =0.14, observed power =0.77) and during passive shoulder range of motion (F_2, 72_ =11.83, *P* < 0.001, partial η^2^ =0.25, observed power =0.99). Significant between-group differences were found in pain during active and passive shoulder range of motion at follow-up (*P* < 0.05) (Fig. 2). The NMES combined with bilateral arm training group had lower pain intensity during active and passive shoulder movement than the TENS combined with bilateral arm training group. Both groups showed significant reductions in pain intensity during shoulder movement after the interventions (*P* < 0.05). However, the NMES combined with bilateral arm training group maintained the improvement in pain reduction during passive shoulder range of motion at follow-up, whereas the TENS combined with bilateral arm training group had increased pain intensity at follow-up compared with post-treatment (*P* = 0.01) (Fig. 2b). These results suggest that the NMES combined with bilateral arm training intervention produced longer-lasting pain relief than TENS combined with bilateral arm training.

**Table 2:** Descriptive and inferential statistics of the primary outcomes (*N* = 38)

	NMES-BAT (*n* = 19)			TENS-BAT (*n* = 19)			Repeated measure ANOVA			
	Pretreatment	Post-treatment	Follow-up	Pretreatment	Post-treatment	Follow-up		*F*	*P*	Partial η^2^
NRS-FRS at rest	0.68 ± 1.46	0 ± 0*	0 ± 0	0.42 ± 0.90	0.21 ± 0.54*	0.42 ± 0.84	Interaction	2.61	0.11	0.07
							group	0.40	0.53	0.01
							time	4.65	0.03*	0.11
NRS-FRS during shoulder AROM	3.89 ± 3.00	0.95 ± 1.18*	0.63 ± 0.83*^#^	3.11 ± 2.16	1.63 ± 1.38*	1.95 ± 1.84*	Interaction	5.84	0.01^#^	0.14
							group	0.69	0.41	0.02
							time	32.60	<0.001*	0.48
NRS-FRS during shoulder PROM	5.79 ± 2.10	2.26 ± 2.05*	2.11 ± 1.79*^#^	5.16 ± 1.54	3.05 ± 1.27*	3.84 ± 2.04*^^^	Interaction	11.83	<0.001^#^	0.25
							group	1.48	0.23	0.04
							time	79.27	<0.001*	0.69
BPI-SF question 3 worst pain	5.95 ± 1.99	2.58 ± 2.17*^#^	2.11 ± 1.76*^#^	5.21 ± 2.18	3.95 ± 1.58*	4.05 ± 2.04	Interaction	10.84	<0.001^#^	0.23
							group	2.61	0.12	0.07
							time	42.19	<0.001*	0.54
BPI-SF question 9 pain interference	1.10 ± 1.20	0.32 ± 0.54*	0.15 ± 0.30*	0.93 ± 0.84	0.44 ± 0.56*	0.42 ± 0.61*	Interaction	1.64	0.21	0.04
							group	0.14	0.71	0.01
							time	20.62	<0.001*	0.36

All data are presented as mean ± SD. Abbreviations: *NMES* neuromuscular electrical stimulation, *TENS* transcutaneous electrical nerve stimulation, *BAT* bilateral arm training, *NRS-FRS* Numerical Rating Scale supplemented with a Faces Rating Scale, *BPI-SF* short form of the Brief Pain Inventory, *AROM* active range of motion, *PROM* passive range of motion

*Significantly different from the pretreatment time point (*P* < 0.05)

^^^Significantly different from the post-treatment time point (*P* < 0.05)

^#^Significantly different from the TENS-BAT group (*P* < 0.05)

**Fig. 3:**
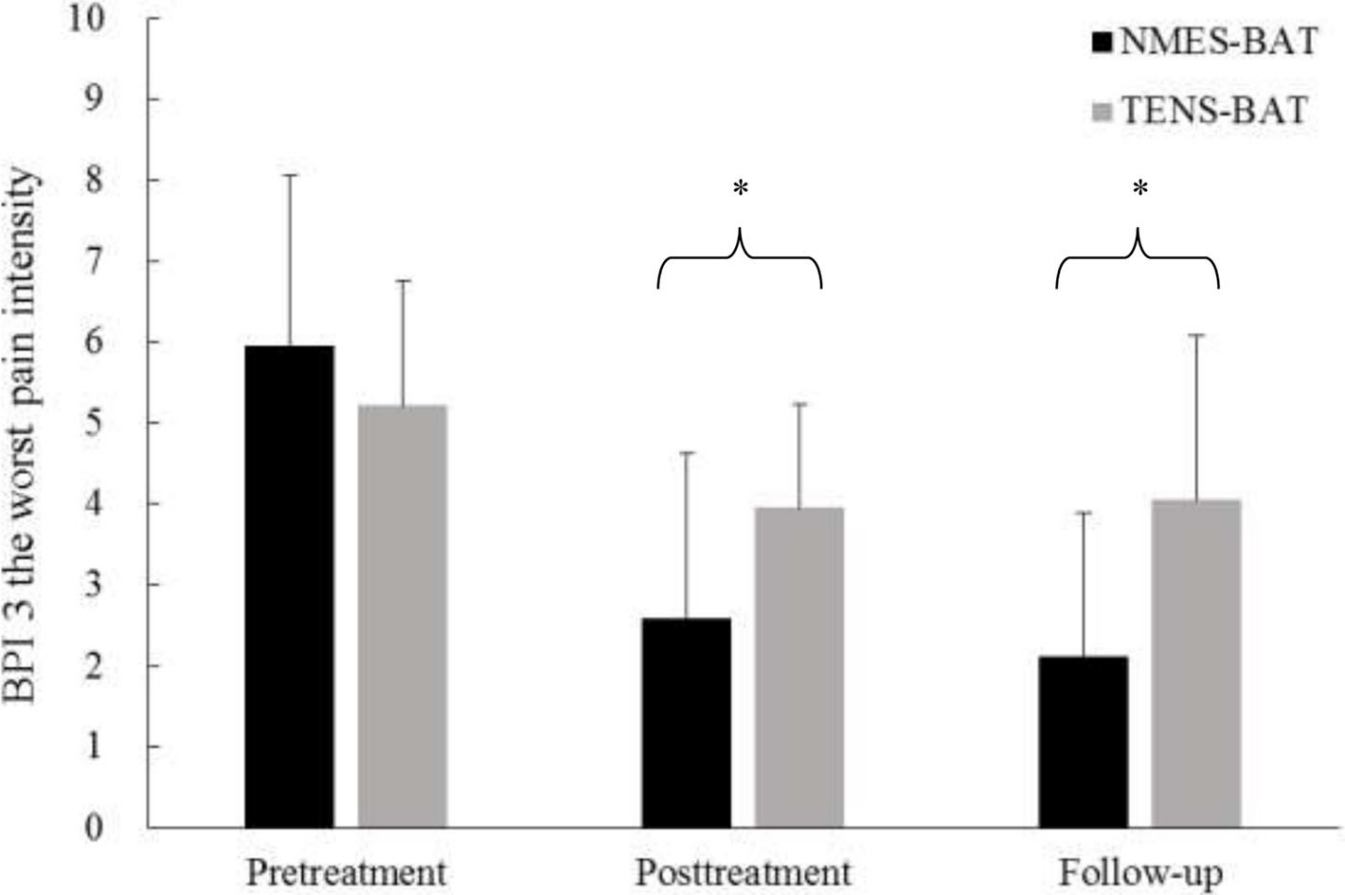
BPI question 3 scores. *P < 0.05 between groups as indicated

There was a significant time-group interaction on question 3 of the BPI-SF (F_1.47, 52.94_ = 10.84, *P <* 0.001, partial η^2^ = 0.23, observed power = 0.96). Both groups showed significant reductions in the worst pain intensity post-treatment, but only the NMES combined with bilateral arm training group maintained pain reduction at follow-up (*P* < 0.05). A significant difference was found between groups on question 3 of the BPI-SF post-treatment and at follow-up (*P* = 0.033 and *P* = 0.003, respectively). The NMES combined with bilateral arm training group had significantly greater reductions in the worst pain than the TENS combined with bilateral arm training group post-treatment and at follow-up (from 5.95 to 2.58 and 2.11 with NMES combined with bilateral arm training vs. from 5.21 to 3.95 and 4.05 with TENS combined with bilateral arm training) (Fig. 3). A significant time effect on question 9 of the BPI-SF for assessing pain interference was found (F_1.27, 45.62_ = 20.62, *P* < 0.001, partial η^2^ = 0.36, observed power = 1.00). Both groups showed significant reductions in pain interference post-treatment and at follow-up (*P* < 0.05) (Table 2).

### Effect of interventions on secondary outcome measures

For total, proximal, and distal scores on the FMA-UL, repeated measures ANOVA showed a significant time effect (total: F_1.27, 45.58_ = 12.60, *P <* 0.001, partial η^2^ = 0.26, observed power = 0.97; proximal: F_1.39, 50.13_ = 12.98, *P <* 0.001, partial η^2^ = 0.27, observed power = 0.98; distal: F_1.23, 44.21_ = 3.88, *P =* 0.05, partial η^2^ = 0.10, observed power = 0.54), but no group-time interaction or group effects (*P* > 0.05) (Table 3). Both groups showed significant improvements in the total score and proximal score of the FMA-UL post-treatment and maintained the improvement at follow-up (*P* < 0.05). There was no significant group effect, but the NMES combined with bilateral arm training group had a numerically larger increase in the total score of upper-limb subscale of the FMA-UL than the TENS combined with bilateral arm training group post-treatment and at follow-up. The mean changes in overall scores from pretreatment to post-treatment and follow-up were 4.06 and 4.37 points (proximal: 3 and 3.27 points; distal: 1.06 and 1.11 points) for the NMES combined with bilateral arm training group and 1.63 and 1.31 points (proximal: 1.21 and 0.89 points; distal: 0.42 and 0.42 points) for the TENS combined with bilateral arm training group, respectively.

**Table 3:** Descriptive and inferential statistics of the secondary outcome measures (*N* = 38)

	NMES-BAT (n = 19)			TENS-BAT (*n* = 19)			Repeated measure ANOVA			
	Pretreatment	Posttreatment	Follow-up	Pretreatment	Posttreatment	Follow-up		*F*	*P*	Partial η^2^
FMA-UL total score (0–66)	41.68 ± 20.17	45.74 ± 17.73*	46.05 ± 17.03*	45.37 ± 17.62	47.00 ± 16.06*	46.68 ± 16.45*	Interaction	3.04	0.08	0.08
							group	0.11	0.74	0.003
							time	12.60	<0.001*	0.26
FMA-UL proximal score (0–42)	29.47 ± 10.35	32.47 ± 7.97*	32.74 ± 7.38*	31.00 ± 7.78	32.21 ± 6.84*	31.89 ± 7.23*	Interaction	3.39	0.06	0.09
							group	0.003	0.96	0.00
							time	12.98	<0.001*	0.27
FMA-UL distal score (0–24)	12.21 ± 10.47	13.26 ± 10.30*	13.32 ± 10.12	14.37 ± 10.26	14.79 ± 9.54*	14.79 ± 9.55	Interaction	0.75	0.42	0.02
							group	0.28	0.60	0.01
							time	3.88	0.05*	0.10
Pain-free passive ROM (degree)										
shoulder abduction	140.26 ± 39.35	162.37 ± 23.42*	171.58 ± 14.73*^#^	121.84 ± 42.63	146.32 ± 34.27*	138.68 ± 35.62*	Interaction	2.93	0.08	0.08
							group	5.23	0.03^#^	0.13
							time	26.37	<0.001*	0.42
shoulder flexion	146.05 ± 34.38	166.84 ± 17.73*	172.11 ± 13.67*	141.84 ± 30.29	159.21 ± 22.44*	154.74 ± 25.36*	Interaction	1.86	0.18	0.05
							group	1.93	0.17	0.05
							time	19.81	<0.001*	0.36
shoulder external rotation	57.63 ± 21.17	70.00 ± 17.40*	74.74 ± 11.96*	55.26 ± 21.44	71.32 ± 15.97*	67.11 ± 17.82*	Interaction	1.79	0.18	0.05
							group	0.32	0.58	0.009
							time	24.25	<0.001*	0.40
shoulder internal rotation	67.63 ± 15.49	72.63 ± 11.83	76.32 ± 10.65*^#^	66.32 ± 21.01	66.32 ± 16.90	62.11 ± 17.10	Interaction	4.74	0.01^#^	0.12
							group	2.57	0.12	0.07
							time	0.85	0.43	0.02

All data are presented as mean ± SD. Abbreviations: *NMES* neuromuscular electrical stimulation, *TENS* transcutaneous electrical nerve stimulation, *BAT* bilateral arm training, *FMA-UL* the upper-limb subscale of the Fugl-Meyer Assessment, *ROM* range of motion

*Significantly different from the pretreatment (*P* < 0.05)

^#^Significantly different from the TENS-BAT group (*P* < 0.05)

There was no significant group-time interaction in pain-free passive shoulder range of motion except for passive shoulder internal rotation (F_2, 72_ = 4.74, *P* = 0.01, partial η^2^ = 0.12, observed power = 0.78) (Table 3). A significant difference was found between groups on passive shoulder internal rotation at follow-up (*P* = 0.004). Moreover, there was a significant group effect on pain-free passive shoulder abduction (F_1, 36_ = 5.23, *P* = 0.03, partial η^2^ = 0.13, observed power = 0.61) (Table 3). The NMES combined with bilateral arm training group had better pain-free passive shoulder abduction than the TENS combined with bilateral arm training group at follow-up. A significant time effect was found in pain-free passive shoulder abduction, flexion, and external rotation (*P* < 0.001). Both groups showed significant improvements in pain-free passive shoulder abduction, flexion, and external rotation post-treatment and at follow-up (*P* < 0.05) (Table 3).

### Comparative effectiveness of interventions in subjects with and without subluxation

Subgroup analyses showed that a significant group effect was identified only for the change in score on the vertical NRS-FRS during active (from pretreatment to follow-up: F_3, 34_ = 3.42, *P* = 0.03) and passive shoulder range of motion (from pretreatment to post-treatment: F_3, 34_ = 3.42, *P =* 0.03; from pretreatment to follow-up: F_3, 34_ = 7.97, *P < 0.001*). NMES combined with bilateral arm training, in patients both with and without subluxation, produced greater reductions in pain during active shoulder movement at follow-up than the TENS combined with bilateral arm training in patients with subluxation (*P =* 0.02; *P =* 0.01, respectively). The NMES combined with bilateral arm training group with subluxation improved more on pain during passive shoulder movement at post-treatment and follow-up than the TENS combined with bilateral arm training group with or without subluxation (*P* < 0.05).

There was no significant group effect on the change in scores on the FMA-UL and or on pain-free passive shoulder flexion, abduction, or external rotation except for shoulder internal rotation (F_3, 34_ = 6.22, *P* = 0.002). Both the NMES combined with bilateral arm training groups with and without subluxation improved more in terms of pain-free range of shoulder internal rotation at follow-up than the TENS combined with bilateral arm training group with subluxation (*P* < 0.001 and *P* = 0.01, respectively). The mean change in overall scores of the FMA-UL from pretreatment to post-treatment was 4.44 and 3.70 points for the NMES combined with bilateral arm training group with and without subluxation, respectively, and 1.88 and 1.45 points for the TENS combined with bilateral arm training group with and without subluxation, respectively.

## Discussion

To our knowledge this is the first randomized controlled study that examined the effects of NMES combined with bilateral arm training on hemiplegic shoulder pain. In support of our hypotheses, this randomized controlled trial provides evidence that the immediate and retained effects of NMES combined with bilateral arm training in reducing pain and improving arm function were better than TENS combined with bilateral arm training for stroke patients with hemiplegic shoulder pain. The results demonstrated that EMG-triggered NMES with bilateral arm training was superior to TENS with bilateral arm training in reducing hemiplegic shoulder pain during shoulder movement, lessening the worst shoulder pain, and increasing pain-free shoulder abduction and internal rotation at follow-up. Both groups improved on pain interference, motor impairment, and pain-free shoulder abduction, flexion, and external rotation.

### Pain reduction in people with stroke

Our results are consistent with previous studies showing positive effects of NMES on pain reduction in stroke patients [12, 14, 16, 18]. Our previous study showed that the smallest real difference in the vertical NRS-FRS for measuring pain after stroke was 1.87 points [30]. In this study, the NMES combined with bilateral arm training group’s scores on the vertical Numerical Rating Scale supplemented with a Faces Rating Scale decreased by 2.94 points during active shoulder range of motion and by 3.53 points during passive shoulder range of motion at post-treatment. The mean changes from pretreatment to post-treatment exceeded the measurement error and were real at the 90% confidence level.

It is noteworthy that the effects of NMES combined with bilateral arm training on pain reduction during movement were maintained for 1 month after the intervention ceased for subjects both with and without subluxation. It is possible that the active participation of the NMES group during the intervention further motivated them to continue using the paretic arm, producing long-lasting effects on pain reduction and pain-free passive shoulder abduction and internal rotation. Conversely, the score on the vertical NRS-FRS in the TENS combined with bilateral arm training group increased at the follow-up assessment, approaching pretreatment levels, especially for subjects with subluxation.

Hemiplegic shoulder pain is a complex phenomenon. Its pathophysiology is not yet fully understood, which results in uncertainty regarding the optimal management strategy for hemiplegic shoulder pain. The cause of hemiplegic shoulder pain is a subject of debate due to its multifactorial etiology. Shoulder subluxation is considered an important risk factor for shoulder pain [3]. In this study, 45% of subjects (17 out of 38) had shoulder subluxation and shoulder pain. Subjects with shoulder subluxation in the EMG-triggered NMES combined with bilateral arm training group had more improvement on pain reduction during shoulder movement than those with subluxation who received TENS combined with bilateral arm training. The participants that improved more on the pain scale were those who received EMG-triggered NMES combined with bilateral arm training, regardless of whether they had subluxation.

Previous studies showed that NMES did not decrease upper-limb pain and improve arm function in patients with acute stroke [13], and that NMES did not improve arm recovery and pain-free passive shoulder external rotation in patients with chronic stroke [17]. In the present study, NMES combined with bilateral arm training significantly relieved shoulder pain and increased the painless range of motion in chronic and subacute stroke patients. Such differences could be attributed to differences in stroke chronicity, measurement tools, treatment dose, and the use of bilateral arm training in this study. The current study recruited subjects much later than the previous studies [13, 16, 18], i.e., after the onset of stroke and development of shoulder pain. It has been proposed that early intervention with NMES in acute stroke may be more beneficial in patients with mild upper-limb impairment, but may interfere with arm recovery in patients with severe impairment [13]. In addition, the most common pain measure used in previous studies was the VAS, which is not recommended for measuring pain after stroke [39]. Finally, the stimulation protocol in this study differed from previous studies in frequency and duration. Investigation of the optimal dosage of the treatment program to maximize the effects of NMES needs to be determined in future research.

Both groups experienced significant reductions in the worst pain intensity post-treatment and pain interference with daily activities. Only the NMES combined with bilateral arm training group maintained the improvement in the worst pain intensity at follow-up. These results were in line with a previous study that showed peripheral nerve stimulation therapy significantly improved pain interference and lessened pain intensity [40]. The retention of the effects of NMES combined with bilateral arm training was similar to that reported in a previous study of intramuscular electrical stimulation in reducing hemiplegic shoulder pain at 12 months post-treatment [41]. Chae et al. defined treatment success as a minimum 2-point reduction in the BPI-SF question 3 post-treatment [41]. In the current study, the mean change in scores on the BPI-SF question 3 from pretreatment to post-treatment in the NMES combined with bilateral arm training group exceeded 2 points (3.37 points), while those in the TENS combined with bilateral arm training group failed to reach this threshold (1.26 points). Given these results, we suggest that short-term EMG-triggered NMES may be a good alternative to long-duration intramuscular electrical stimulation for inducing voluntary contraction and providing pain relief.

### Advantages of bilateral arm training

In the current study, both groups improved significantly on the FMA-UL. Lin et al. found that bilateral arm training significantly reduced motor impairment of the affected upper limb in stroke patients [42]. Bilateral arm training comprises repetitive bilateral arm movements in symmetrical or alternating patterns [43] by which participants learn to shape the movement trajectory of the affected arm as symmetrically as the non-affected arm. Bilateral arm training can induce concurrent activation of the ipsilateral tracts, cortical disinhibition, and other neural cross-talk, resulting in improved motor control in the affected limb [43]. Thus, bilateral arm training has been proposed to be a potential rehabilitation intervention for upper-limb hemiparesis, especially in the proximal region [42].

### Benefits of EMG-triggered NMES with bilateral arm training

Although there was no significant between-group difference on the FMA-UL, the mean changes in the total score of the FMA-UL from pretreatment to post-treatment and follow-up were 4.06 and 4.37 points for the NMES combined with bilateral arm training group and 1.63 and 1.31 points for the TENS combined with bilateral arm training group, respectively. The estimated minimal clinically important difference on the total score of the FMA-UL in patients with chronic stroke is 4.25 points, and only the NMES combined with bilateral arm training group experienced changes close to this threshold [44]. The improvement of the total score of the FMA-UL after NMES combined with bilateral arm training was mainly on the proximal score of the FMA-UL (3 and 3.27 points from pretreatment to post-treatment and follow-up, respectively). The benefit on the upper-limb subscale of the FMA-UL was probably attributed to an augmented intervention effect of NMES and bilateral arm training. EMG-triggered NMES can augment movement of the hemiparetic arm, increase cognitive attention through proprioceptive sensory feedback, and may enhance arm function in stroke patients [45]. Bilateral arm training was reported to produce greater gains in the proximal score of the FMA-UL than a control intervention in patients with chronic stroke [46]. The additional facilitation in the affected arm is probably because concurrent activation of both arms facilitates intracortical activity and decreases inhibition in both hemispheres [47]. Given this perspective, it is hypothesized that repetitive muscle contraction and cognitive involvement in generating repetitive movements are critical for redeveloping spontaneous motor control [48].

### Limitations

Limitations of this study were a lack of placebo control and the short duration of follow-up. Perhaps a longer period of evaluation would have generated proportionately different outcomes between the treatment groups. Moreover, research that compares the intervention effects of EMG-triggered NMES combined with bilateral arm training, EMG-triggered NMES only, and bilateral arm training only would provide more insight into the benefits of the combined therapy.

## Conclusions

EMG-triggered NMES combined with bilateral arm training was better than TENS with bilateral arm training for reducing hemiplegic shoulder pain during movement, lessening the worst shoulder pain, and improving pain-free shoulder abduction and internal rotation for stroke patients with hemiplegic shoulder pain. Such improvements appear to be sustained beyond the immediate time frame of the treatment.

## Abbreviations

BPI-SF: Short form of the brief pain inventory
FMA-UL: Upper-limb subscale of the Fugl-Meyer Assessment
NMES: Neuromuscular electrical stimulation
NRS-FRS: Numerical rating scale supplemented with a faces rating scale
TENS: Transcutaneous electrical nerve stimulation

## References

[1] Lindgren I, Jonsson AC, Norrving B, Lindgren A (2007). Shoulder pain after stroke: a prospective population-based study. Stroke.

[2] Sheffler LR, Chae J (2007). Neuromuscular electrical stimulation in neurorehabilitation. Muscle Nerve.

[3] Viana R, Pereira S, Mehta S, Miller T, Teasell R (2012). Evidence for therapeutic interventions for hemiplegic shoulder pain during the chronic stage of stroke: a review. Top Stroke Rehabil.

[4] Snels IA, Dekker JH, van der Lee JH, Lankhorst GJ, Beckerman H, Bouter LM (2002). Treating patients with hemiplegic shoulder pain. Am J Phys Med Rehabil..

[5] Lakse E, Gunduz B, Erhan B, Celik EC (2009). The effect of local injections in hemiplegic shoulder pain: a prospective, randomized, controlled study. Am J Phys Med Rehabil.

[6] de Kroon JR, Ijzerman MJ, Chae J, Lankhorst GJ, Zilvold G (2005). Relation between stimulation characteristics and clinical outcome in studies using electrical stimulation to improve motor control of the upper extremity in stroke. J Rehabil Med.

[7] Chae J, Sheffler L, Knutson J (2008). Neuromuscular electrical stimulation for motor restoration in hemiplegia. Top Stroke Rehabil.

[8] Price CI, Pandyan AD (2001). Electrical stimulation for preventing and treating post-stroke shoulder pain: a systematic Cochrane review. Clin Rehabil.

[9] Doucet BM, Lam A, Griffin L (2012). Neuromuscular electrical stimulation for skeletal muscle function. Yale J Biol Med.

[10] IJzerman MJ, Renzenbrink GJ, Geurts AC (2009). Neuromuscular stimulation after stroke: from technology to clinical deployment. Expert Rev Neurother.

[11] Baker LL, Parker K (1986). Neuromuscular electrical stimulation of the muscles surrounding the shoulder. Phys Ther.

[12] Chantraine A, Baribeault A, Uebelhart D, Gremion G (1999). Shoulder pain and dysfunction in hemiplegia: effects of functional electrical stimulation. Arch Phys Med Rehabil.

[13] Church C, Price C, Pandyan AD, Huntley S, Curless R, Rodgers H (2006). Randomized controlled trial to evaluate the effect of surface neuromuscular electrical stimulation to the shoulder after acute stroke. Stroke.

[14] Kobayashi H, Onishi H, Ihashi K, Yagi R, Handa Y (1999). Reduction in subluxation and improved muscle function of the hemiplegic shoulder joint after therapeutic electrical stimulation. J Electromyogr Kinesiol.

[15] Koyuncu E, Nakipoglu-Yuzer GF, Dogan A, Ozgirgin N (2010). The effectiveness of functional electrical stimulation for the treatment of shoulder subluxation and shoulder pain in hemiplegic patients: a randomized controlled trial. Disabil Rehabil.

[16] Linn SL, Granat MH, Lees KR (1999). Prevention of shoulder subluxation after stroke with electrical stimulation. Stroke.

[17] Wang RY, Yang YR, Tsai MW, Wang WT, Chan RC (2002). Effects of functional electric stimulation on upper limb motor function and shoulder range of motion in hemiplegic patients. Am J Phys Med Rehabil..

[18] Faghri PD, Rodgers MM, Glaser RM, Bors JG, Ho C, Akuthota P (1994). The effects of functional electrical stimulation on shoulder subluxation, arm function recovery, and shoulder pain in hemiplegic stroke patients. Arch Phys Med Rehabil.

[19] Kalichman L, Ratmansky M (2011). Underlying pathology and associated factors of hemiplegic shoulder pain. Am J Phys Med Rehabil..

[20] Ratnasabapathy Y, Broad J, Baskett J, Pledger M, Marshall J, Bonita R (2003). Shoulder pain in people with a stroke: a population-based study. Clin Rehabil.

[21] Mangold S, Schuster C, Keller T, Zimmermann-Schlatter A, Ettlin T (2009). Motor training of upper extremity with functional electrical stimulation in early stroke rehabilitation. Neurorehabil Neural Repair.

[22] Chan MK, Tong RK, Chung KY (2009). Bilateral upper limb training with functional electric stimulation in patients with chronic stroke. Neurorehabil Neural Repair.

[23] Cauraugh JH, Kim S (2002). Two coupled motor recovery protocols are better than one: electromyogram-triggered neuromuscular stimulation and bilateral movements. Stroke.

[24] Cauraugh JH, Kim SB, Duley A (2005). Coupled bilateral movements and active neuromuscular stimulation: intralimb transfer evidence during bimanual aiming. Neurosci Lett.

[25] Basmajian JV, Bazant FJ (1959). Factors preventing downward dislocation of the adducted shoulder joint. An electromyographic and morphological study. J Bone Joint Surg Am.

[26] Paci M, Nannetti L, Rinaldi LA (2005). Glenohumeral subluxation in hemiplegia: an overview. J Rehabil Res Dev.

[27] Delagi EF, Perotto AO, Iazzetti J, Morrison D, Perotto AO, Springfield IL, Thomas CC (2011). Shoulder joint. Anatomical guide for the Electromyographer: the limbs and trunk.

[28] Aras MD, Gokkaya NK, Comert D, Kaya A, Cakci A (2004). Shoulder pain in hemiplegia: results from a national rehabilitation hospital in Turkey. Am J Phys Med Rehabil..

[29] Murie-Fernandez M, Carmona Iragui M, Gnanakumar V, Meyer M, Foley N, Teasell R (2012). Painful hemiplegic shoulder in stroke patients: causes and management. Neurologia.

[30] Chuang LL, CY W, Lin KC, Hsieh CJ (2014). Relative and absolute reliability of a vertical numerical pain rating scale supplemented with a faces pain scale after stroke. Phys Ther.

[31] Cleeland CS, Ryan KM (1994). Pain assessment: global use of the brief pain inventory. Ann Acad Med Singap.

[32] Ger LP, Ho ST, Sun WZ, Wang MS, Cleeland CS (1999). Validation of the brief pain inventory in a Taiwanese population. J Pain Symptom Manag.

[33] Wang XS, Mendoza TR, Gao SZ, Cleeland CS (1996). The Chinese version of the brief pain inventory (BPI-C): its development and use in a study of cancer pain. Pain.

[34] Fugl-Meyer AR, Jaasko L, Leyman I, Olsson S, Steglind S (1975). The post-stroke hemiplegic patient. 1. A method for evaluation of physical performance. Scand J Rehabil Med.

[35] Duncan PW, Propst M, Nelson SG (1983). Reliability of the Fugl-Meyer assessment of sensorimotor recovery following cerebrovascular accident. Phys Ther.

[36] Bohannon RW, Larkin PA, Smith MB, Horton MG (1986). Shoulder pain in hemiplegia: statistical relationship with five variables. Arch Phys Med Rehabil.

[37] Faul F, Erdfelder E, Lang AG, Buchner A (2007). G*power 3: a flexible statistical power analysis program for the social, behavioral, and biomedical sciences. Behav Res Methods.

[38] Bohannon RW, Andrews AW (1990). Shoulder subluxation and pain in stroke patients. Am J Phys Med Rehabil..

[39] Price CI, Curless RH, Rodgers H (1999). Can stroke patients use visual analogue scales?. Stroke.

[40] Wilson RD, Gunzler DD, Bennett ME, Chae J (2014). Peripheral nerve stimulation compared with usual care for pain relief of hemiplegic shoulder pain: a randomized controlled trial. Am J Phys Med Rehabil..

[41] Chae J, DT Y, Walker ME, Kirsteins A, Elovic EP, Flanagan SR, Harvey RL, Zorowitz RD, Frost FS, Grill JH, Fang ZP (2005). Intramuscular electrical stimulation for hemiplegic shoulder pain: a 12-month follow-up of a multiple-center, randomized clinical trial. Am J Phys Med Rehabil.

[42] Lin KC, Chen YA, Chen CL, CY W, Chang YF (2010). The effects of bilateral arm training on motor control and functional performance in chronic stroke: a randomized controlled study. Neurorehabil Neural Repair.

[43] Stoykov ME, Corcos DM (2009). A review of bilateral training for upper extremity hemiparesis. Occup Ther Int.

[44] Page SJ, Fulk GD, Boyne P (2012). Clinically important differences for the upper-extremity Fugl-Meyer scale in people with minimal to moderate impairment due to chronic stroke. Phys Ther.

[45] Wu FC, Lin YT, Kuo TS, Luh JJ, Lai JS (2011). Clinical effects of combined bilateral arm training with functional electrical stimulation in patients with stroke. IEEE Int Conf Rehabil Robot.

[46] Lin KC, Chang YF, CY W, Chen YA (2009). Effects of constraint-induced therapy versus bilateral arm training on motor performance, daily functions, and quality of life in stroke survivors. Neurorehabil Neural Repair.

[47] McCombe Waller S, Forrester L, Villagra F, Whitall J (2008). Intracortical inhibition and facilitation with unilateral dominant, unilateral nondominant and bilateral movement tasks in left- and right-handed adults. J Neurol Sci.

[48] Cauraugh JH, Kim SB (2003). Chronic stroke motor recovery: duration of active neuromuscular stimulation. J Neurol Sci.

